# Formation of social and household skills in children with hand defects

**DOI:** 10.1007/s11832-015-0668-6

**Published:** 2015-08-05

**Authors:** Nataly Klimon, Alexander Koryukov, Nina Loseva, Elena Starobina

**Affiliations:** St. Petersburg Orthopaedic Center of Expertise, Prosthetics and Rehabilitation of Disabled, Bestuzaevskay Str, 50, St. Petersburg, 195067 Russia; St. Petersburg Institute Expertise of Medical Doctors Advance, Big Mariinsky Prospekt, 11, St. Petersburg, Russia

**Keywords:** Children with hand defects, Social and household skills, Types of grip, Functional capacity of a hand to grip, Social rehabilitation, Play therapy

## Abstract

**Purpose:**

The aim of this study was to consider the peculiarities of forming social and household skills, and the criteria for their evaluation, as well as an assessment of functional capacity, in children with hand defects both before and after surgical treatment and rehabilitation courses using a system of games.

**Methods:**

We elaborated and implemented a program of social rehabilitation of preschool children with congenital and acquired hand defects for the development of their functional capabilities and the formation of social and household skills after surgical treatment and prosthetics using play therapy methods. As part of this work, 140 preschool children aged 3–7 years underwent social rehabilitation. Most of the children had congenital hand defects—122 children (87 %): 96 children (79 %) with ectrodactylia, adactylia, hypoplasia, aplasia, hand splitting, club hand, or partial gigantism; 26 children (21 %) with congenital syndactylism and constricted bonds and 18 children (13 %) with acquired defects (burn deformity, amputation). 110 children (79 %) had reached the stage of surgical correction; 30 children (21 %) reached the stage of prosthetics. Most of the children participating in the experiment (78 children, 56 %) had defects of fingers on one hand. The program aimed at solving specific rehabilitation tasks: formation and improvement of all possible types of grip under the existing defect including those after surgery and prosthetics; development of tactile sensations in fingers; development of fine motor skills; increase in range of motion in all joints of the damaged hand; development of attention and concentration; formation of social and household skills appropriate to age; and development of the ability to achieve the set task.

**Results:**

Analysis of the level of social and household skills of children with hand defects undergoing rehabilitation treatment at the hospital depending on the age prior to medical and social rehabilitation showed that preschool children with hand defects in the age category of 3 years demonstrated the highest results in the level of social and household skills (31 %) as compared with children in other age categories. The indicators for children aged 4 and 5 years were slightly lower, 25 and 26 %, respectively. The lowest values were recorded among children aged 6: 20 %.

**Conclusion:**

Statistically significant parameters of the level of functional capacity of hand grip and social and household skills in children with hand defects obtained in the course of the investigation indicated that the use of play therapy measures significantly increased the effect of medical treatment irrespective of the type of defect. These data indicate that play therapy measures given immediately after surgery or prosthetics can significantly increase the efficiency of rehabilitation even in its early stages.

## Introduction

The capacity to operate the entire range of social and household skills necessary for self-care and daily activities plays an important role in the process of social adaptation of children with hand defects [8].

Hand defects of congenital and post-traumatic etiology significantly reduce the functional capabilities of a child, and the lack of the necessary initial social experience makes it impossible to acquire socially significant skills and requires special measures for their formation.

The hand injuries most common among preschool children are caused by injuries with sharp objects, injured tissues, thermal injuries, and injuries with electric current, and account for more than 30 % of all visits to ambulance and orthopedics centres, often leading to amputation of fingers at different levels [9].

Pronounced anatomical, functional and biomechanical alterations in the case of hand defects completely or partially impair functional capacity, and limit the formation of social and household skills. In addition, these pronounced cosmetic defects have a negative effect on the psychological status of the child.

Children of preschool age with complex defects, particularly defects of both hands, are subject to medical rehabilitation at several stages. At the same time, the motor capabilities achieved at an early stage of surgical treatment undergo significant changes at subsequent stages.

The child is growing and developing, the compensatory mechanisms developed by them are changing, and the final level of motor habits achieved at an early stage of correction becomes the main one in the planning of subsequent stages. In such a situation, the diversity and variability of the forms and methods of treatment which can be used is extremely important [1, 4, 5].

The purpose of the work with children with hand defects should be their purposeful preparation for life, a decreasing level of care from other people as well as achievement of the maximum possible level of independence.

Since the leading type of activity in preschool children is games, the rehabilitation methods for the development of major age-appropriate skills and abilities should be applied in the course of play therapy. The aim of play therapy is to help the child to achieve the required level of skills in self-care and social adaptation corresponding to their age, using games and a well-organized developmental environment.

Due to the fact that periodic stays at or outpatient visits to the rehabilitation centre by the child and their family members are always accompanied by unavoidable breaks, during which the child in time loses the skills acquired by them at the rehabilitation centre, the parents should be trained in basic techniques of home rehabilitation [3].

It is the family which from an early age should be engaged in development of the child’s social and household skills which will further promote their socialization.

Mastering of the diverse skills and abilities necessary in everyday life and their improvement consists not only in the fact that the child begins to manage without the help of an adult [6]. They develop independence, the ability to overcome difficulties, and the ability of conation. Independence, in its turn, often improves the overall organization of behaviour.

It is the parents who set an example of action, approve the correct result, and point to the errors, at the same time teaching the preschooler to control and evaluate their actions and compare them with the example.

Unfortunately, in practice, low motivation of both parents and the child is often observed [7]. This, in its turn, leads to care and assistance needed by the parents, which significantly complicates further adaptation of the child.

## Materials and methods

We elaborated and implemented a program of social rehabilitation of preschool children with congenital and acquired hand defects for the development of their functional capabilities and formation of social and household skills after surgical remediation and prosthetics using a course of play therapy.

The program aims at solving specific rehabilitation tasks:

- formation and improvement of all possible types of grip under the existing defect including those after surgery and prosthetics;
- development of tactile sensations in fingers;
- development of fine motor skills;
- increase in range of motion in all joints of the damaged hand;
- development of attention and concentration;
- formation of social and household skills appropriate to age;
- development of the ability to achieve the set task.

When determining the purpose of the game activities in developing social and household skills while drawing up a plan of correctional actions, the level of social and household skills in the children was taken into account, together with their compliance with the age norm and the level of the hand’s functional capacity to grip. When systematizing the games and play materials for the development of social and household skills, account was taken of the child’s age, the severity of the defect, and the capacity of the hand to perform different types of grip.

The development of an individual program in each case was carried out at the time of primary diagnosis and adjusted depending on the child’s age, the level of their psychophysical development, and the defect, and included the following stages:

1. Diagnostic stage (carried out using games):
  assessment of the child’s capabilities prior to surgery and after it;
  drawing up a program of improving the functionality of the hand, and formation/development of social and household skills of the child after medical remediation taking into account the main type of activity.

The diagnostic tools are based on the games used for evaluating different types of grip and social and household skills which are borrowed from the course of play therapy developed by the authors of the paper.

2. Organization of remediation and developing environment:
  selection of play materials taking into account the age of the child and the functional capacity of the hand to grip;
  observance of sanitary hygiene requirements to conditions of teaching children with hand defects.
3. Remedial and development training of children aimed at improving the functionality of the hand and formation/development of social and household skills;
4. Improvement of psychological, pedagogical and social competence of the parents;
5. Assessment of the efficiency of remedial and development training with the use of play therapy measures.
6. Drawing up of an intervention program under home conditions focusing on the zone of proximal development.

Diagnosis of functional capacity of the hand was carried out individually in accordance with the child’s age, level of mental and physical development before and after treatment (surgery and prosthetics), and also the course of the play therapy also.

**Fig. 1:**
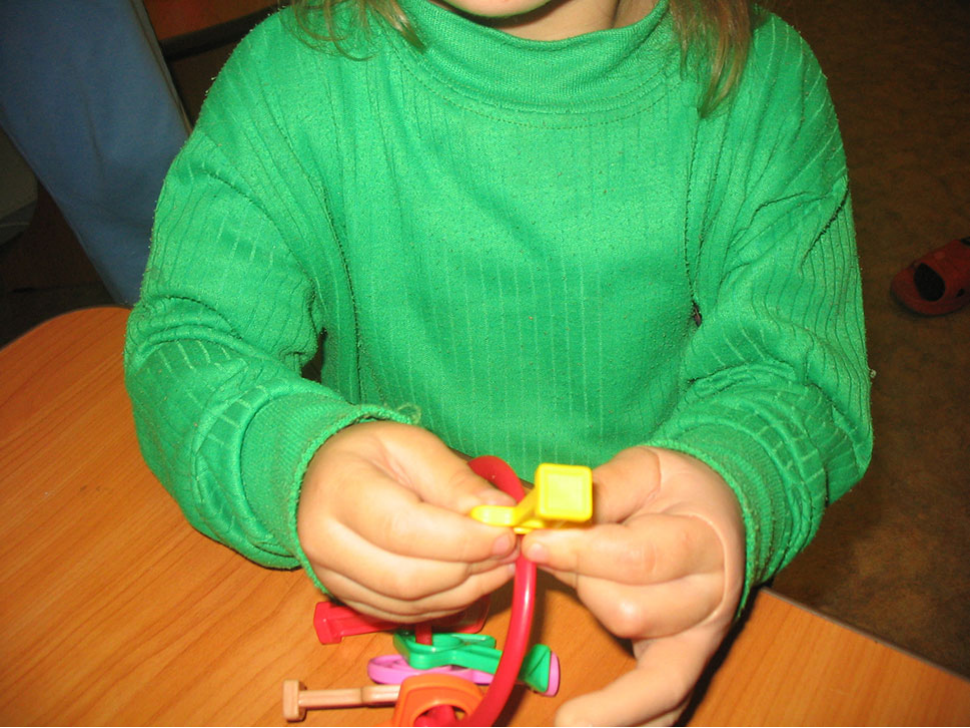
End grip with fingertips

**Fig. 2:**
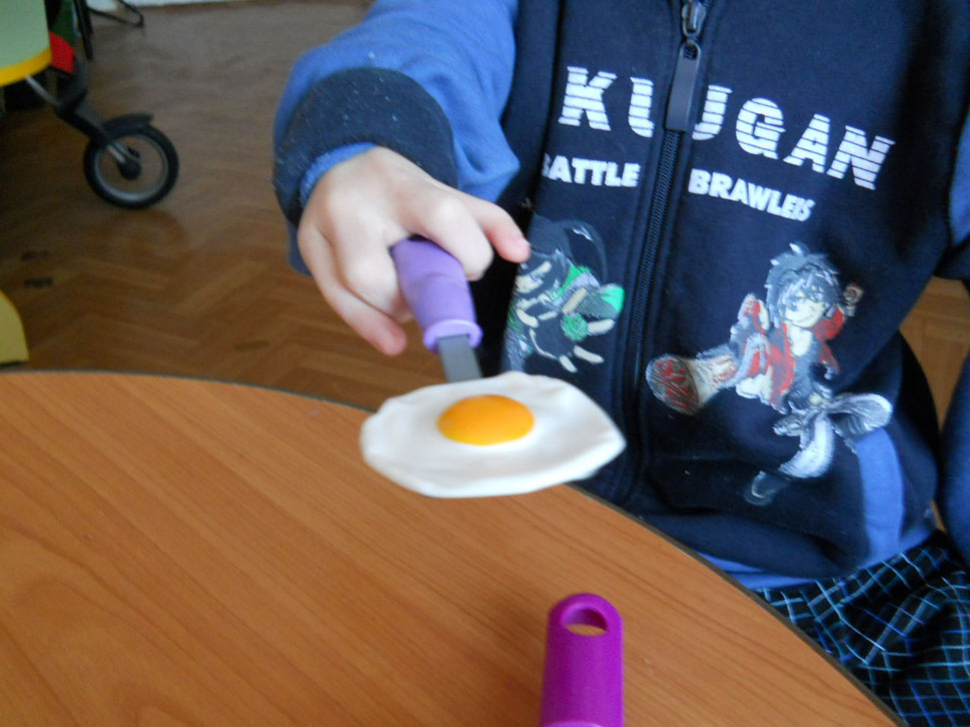
Lateral grip

**Fig. 3:**
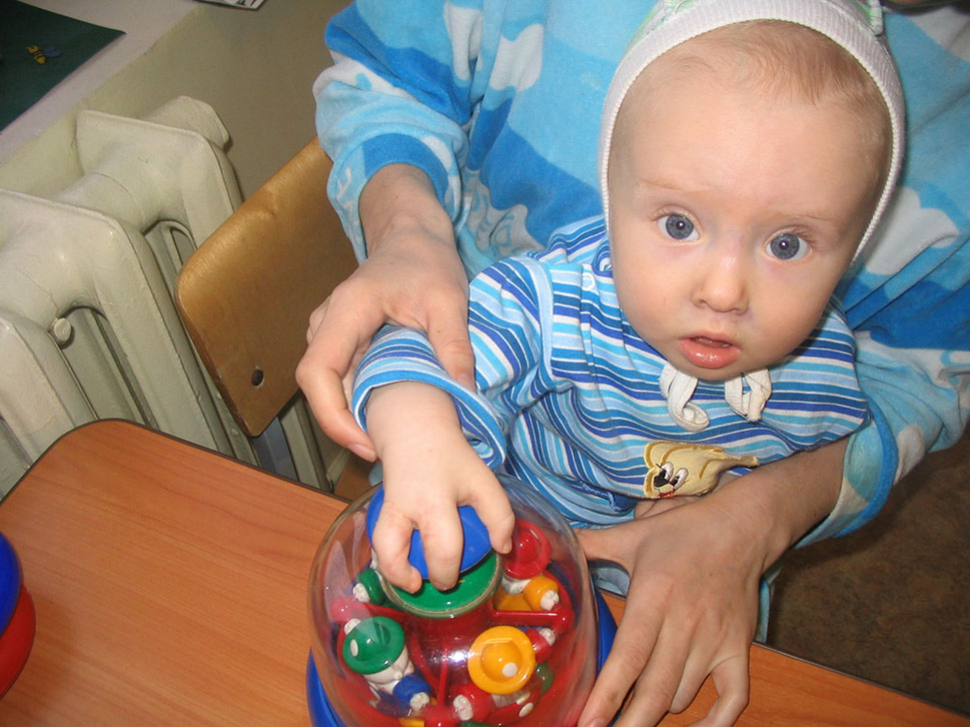
Form-shaping grip

**Fig. 4:**
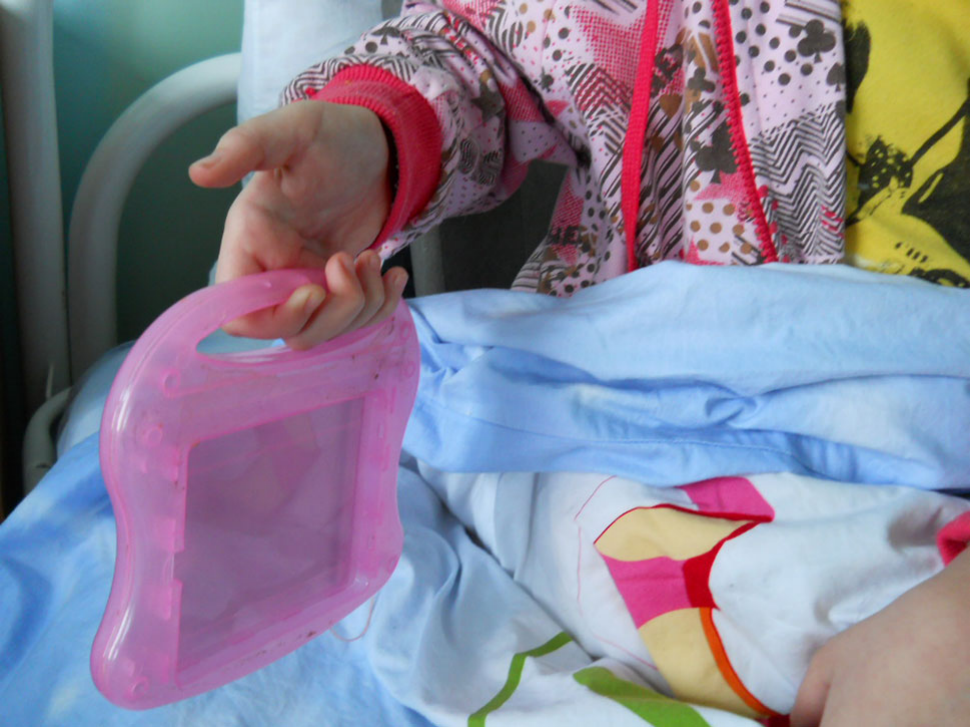
Hooking grip

The evaluation of hand function was performed on the basis of four main types of grip: end (Fig. 1), lateral (Fig. 2), form-shaping (Fig. 3) and hooking (Fig. 4), and was accomplished using a specially designed 4-point scale:

- 0 points—the child cannot take and hold a toy;
- 1 point—the child can take and hold a toy with great difficulty;
- 2 points—the child can take and hold a toy with minor difficulty;
- 3 points—the child can freely take and hold a toy.

After assessing all types of grip, an overall assessment of the functional capacity of a hand to grip was made in children with congenital and acquired hand defects by applying a specially developed formula.

The final result of the functional capacity of the hand to grip in children was presented as a percentage.

The level of functional capacity of the hand to grip was assessed as follows:

- High functional capacity of the hand to grip;
- Average functional capacity of the hand to grip;
- Low functional capacity of the hand to grip.

Assessment of hand function in children was carried out in the form of a game familiar to them using the game tools and toys of various sizes, taking into account the child’s age and the defect of their hand, which allowed a more accurate assessment of the gripping function for making a decision on the choice of a complex of remedial and development training.

During evaluation, the child was offered the task appropriate to their biological age. If they could not cope with it, the teacher picked the task that is assigned to an earlier age. This was considered to be the level of functional ability of the hand at the time of evaluation.

For example, estimating the end grip used games with the pyramid, “lacing”, etc.; lateral—“clockwork toys”, “house of young animals”; shaping—“jolly ball”, “navigate a ball”; and hooking—“roll the toy” “bring your house”.

This allowed a more accurate assessment of the hand grip for a decision on the choice of a complex correction and knowledge of development.

On the basis of analyzing the program of development and education of children at kindergartens, “Krokha (tiny one)”, “Childhood”, “Development and education in a kindergarten”, recommended by the Ministry of Education of the Russian Federation, the authors of the paper drew up a list of the main social and household skills and types of grips used for accomplishing them which are the age norm for preschool children aged 3, 4, 5, and 6. These data are presented in Table 1.

**Table 1:** List of social and household skills and types of grip used for accomplishing them by preschool children of different age as a norm

Age	Social and household skills	Type of grip
3 years	Washes up on their own, washes their hands	Lateral
	Brushes teeth	Form-shaping
	Uses a comb	Form-shaping
	Uses a handkerchief	End
	Eats on their own, holds a spoon correctly, can use a fork	Lateral
	Can pour milk or water into a cup and a plate	Lateral
	Dresses and undresses on their own with assistance of an adult	End Form-shaping
	Can button and unbutton clothes, open snap fasteners with assistance of an adult	End
	Can zip and unzip a zipper	End, lateral
	Fastens and unfastens shoe laces with assistance of an adult	End
	Freely turns a door handle	Lateral
	Opens and closes a water tap	Form-shaping
	Gathers toys with assistance of an adult	All types of grips
4 years	Eats on their own	End
	Correctly uses tableware (fork, spoon, knife)	End Lateral
	Uses a napkin on their own	Form-shaping
	Fastens shoe laces on their own	End
	Lays the table, collects dishes and washes up after meals	All types of grips
	Dusts, sweeps the floor, collects the toys	All types of grips
	Makes the bed after sleeping with assistance of an adult	All types of grips
5 years	Virtually does everything on their own	All types of grips
	Masters the techniques of cleaning footwear	Form-shaping
	Masters the techniques of elementary first aid in case of trauma (applies iodine, bandages a finger)	End
	Makes the bed on their own after sleeping	All types of grips
6 years	Can thread a needle	End
	Can sew on buttons	End
	Can water flowers	Form-shaping
	Can sow small and large seeds	End
	Boys can use carpentry tools (hammer, saw)	All types of grips
	Girls can wash, iron doll’s clothes	All types of grips
	Can wash toys, perform minor repairs of toys as well as of books, clothes for dolls	All types of grips

Diagnosis of the level of social and household skills in children with hand defects was made individually, taking into account the age features for each skill separately, using the following system of points:

- 0 points—skill is not performed;
- 1 point—skill is performed with great difficulty, slowly;
- 2 points—skill is performed with minor difficulties;
- 3 points—skill is performed without difficulties.

The total score (depending on age) obtained in the course of diagnosis showed the level of social and life skills at the time of the study.

On the basis of the criteria developed, the developing compensatory mechanisms used by the child in everyday life were revealed; study of their new functional capacities after surgery was conducted as well as study of the changes in the quality of life of the child after the remedial training. Age features and development level of the child were taken into account.

The start of corrective exercises after medical rehabilitation was determined individually depending on the outcome of surgery, the nature of disease, the severity of motor impairment and the level of social and living skills [2]. The correctional program consisted of the following sessions: in the hospital (an average of 5–10 sessions), and at home. The sequence of activities is based on the principle: “from simple actions to complex”. The lesson duration is from 5 to 30 min every day. The number of games used is gradually increased—from 3 to 7–8.

140 preschool children aged 3–7 years were involved in the experimental work. 70 of them completed the full course of medical rehabilitation and play therapy (experimental group) and 70 preschoolers received only a course of medical rehabilitation—exercise therapy, physiotherapy, and massage (control group).

The participants comprised 74 boys (53 %) and 66 girls (47 %). Ten of them visited general-type kindergartens (7 %); 14, a specialized kindergarten (10 %); 116 (83 %) children did not visit any children’s institutions. 102 children (73 %) lived in two-parent families and 32 children (23 %) in single-parent families; 6 children (4 %) lived with a guardian.

Most of the children had congenital hand defects—122 children (87 %): 96 children (79 %) had ectrodactylia, adactylia, hypoplasia, aplasia, hand splitting, club hand or partial gigantism; 26 children (21 %) had congenital syndactylism or constricted bonds; 18 children (13 %) had acquired defects (burn deformity, amputation). 110 children (79 %) had reached the stage of surgical correction; 30 children (21 %) the stage of prosthetics. Most of the children participating in the experiment had defects of the fingers on one hand—78 children (56 %).

The distribution of children depending on type of defect in the control and experimental groups was similar and is presented in Table 2.

**Table 2:** Distribution of children depending on the type of defect in the control and experimental groups

No.	Type of defect	Control group, *n* (%)	Experimental group, *n* (%)
1	Congenital hand defects (ectrodactylia, adactylia, hypoplasia, aplasia etc.)	48 (69 %)	48 (69 %)
2	Congenital syndactylism and congenital constricted bonds	13 (19 %)	13 (19 %)
3	Acquired hand defects (burn deformity, amputation)	9 (13 %)	9 (13 %)
	Total	70 (100 %)	70 (100 %)

## Results

Analysis of the level of social and household skills of children with hand defects undergoing rehabilitation treatment at the hospital depending on age prior to medical and social rehabilitation showed that preschool children with hand defects in the age category of 3 years had the highest result in the level of social and household skills (31.0 %) as compared with children of other age categories. The indicators for children of 4 and 5 years were slightly lower, 25 and 26 %, respectively. The lowest values of the investigated parameter were recorded among children aged 6: 21 %.

Limitation of social and household skills in children with hand defects was worsened by a low functional capacity of the hand to grip, which showed up as a slow rate of performing actions, in insufficient precision of movements of the hand and individual fingers, and in difficulties encountered when gripping objects.

**Fig. 5:**
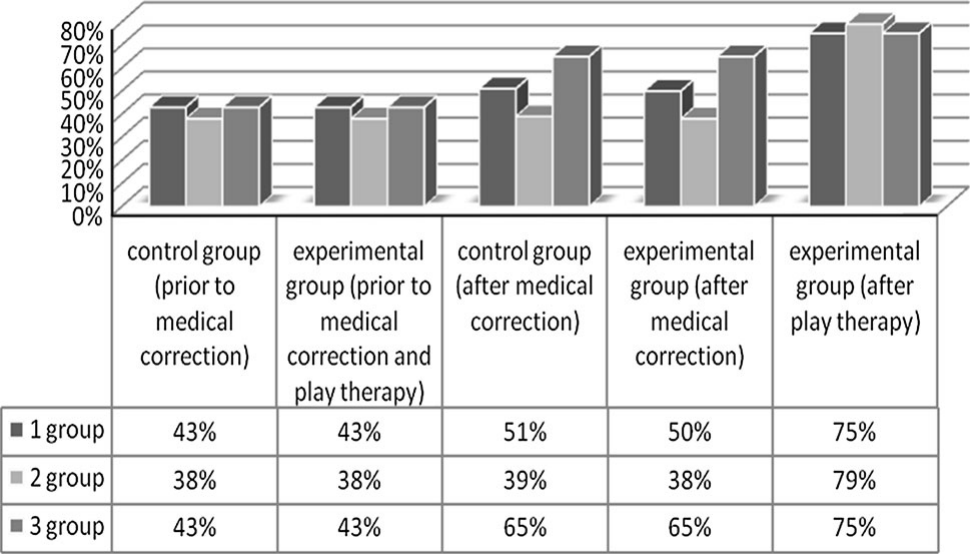
Comparative data on the level of functional ability of the hand prior to and after medical correction and after play therapy

The initial level of functional ability of the hand to grip in children with congenital hand defects (ectrodactylia, adactylia, hypoplasia, aplasia, etc.) and in children with acquired defects (burn deformity, amputation) was similar, at 43 % (Fig. 5). Lower values of the investigated parameter were recorded in children with syndactylism and congenital constricted bonds, at 38 %.

After medical correction (reconstructive surgery, prostheses, etc.) in children with hand defects there was a significant improvement in the functional ability of the hand to grip, both in the control and the experimental groups. After the course of play therapy, children in the experimental group showed significant increases in the functional capacity of the hand to grip.

Children in the experimental group with congenital hand defects (ectrodactylia, adactylia, hypoplasia, aplasia, etc.) and children with acquired defects (burn deformity, amputation) displayed similar changes after medical correction and a course of play therapy in the level of functional ability of the hand to grip; it increased from 43 to 75 % (significant difference *p* ≤ 0.05). In the experimental group of children with congenital syndactylism and congenital constricted bonds the highest growth of the investigated parameter was recorded, from 38 % to 79 % (*p* ≤ 0.05). In the control group, where the corrective actions were taken only within the framework of medical rehabilitation (exercise therapy, physiotherapy, massage), regardless of diagnosis, the changes were not significant.

**Fig. 6:**
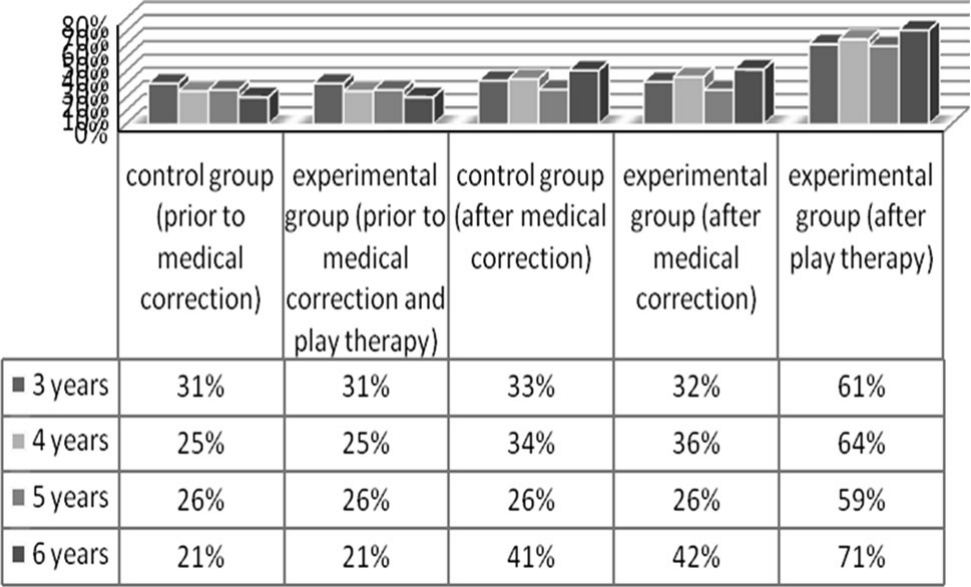
Comparative indicators of the level of social and household skills in children with hand defects depending on age prior to and after medical correction and after play therapy

Significant changes in the level of functional ability of the hand to grip after medical correction and play therapy increased the level of social and household skills (Fig. 6).

Comparative analysis of the level of social and household skills in children with hand defects prior to medical correction and after play therapy showed that in experimental groups of all ages there were significant changes in the level of social and household skills:

- in the age group of 6-year-old preschoolers the maximum increase in the level of social and household skills was recorded, from 21 to 71 %, whereas in the control group it was to 41 %;
- a slightly lower increase was recorded in the age group of 4 years, from 25 to 64 % whereas in the control group it was to 34 %;
- approximately similar changes in the level of social and household skills were recorded in the age of the experimental groups of 3 and 5 years, respectively, from 31 to 61 % and from 26 to 59 %, while in the control groups these parameters as compared with the initial ones changed in children of 3 years from 31 to 33 % and in 5-year-old stayed at the same level: 26 %.

## Conclusion

Statistically significant indicators of the level of functional ability of the hand to grip and social and household skills in children with hand defects recorded in the course of the study indicate that the use of play therapy significantly increases the effect of medical treatment regardless of the type of defect. These data may indicate that a course of play therapy with the use of the games system after surgery or prosthesis significantly increases the efficiency of rehabilitation in its early stages.

In the course of this work it was revealed that performing the tasks of the game proposed with regard for motivation, the children obtained pleasure with a low fatigability threshold, and more quickly and successfully adapted to their new abilities and to the environment through the play activity, which is natural for them.

The main condition of the effective activity is its personal meaning for the child. If an active participation of a child in their own rehabilitation is necessary, the main task is to create all the conditions for motivating the child for the type of activity, which is necessary from the viewpoint of the general purposes of their rehabilitation.
